# Ion Torrent sequencing as a tool for mutation discovery in the flax (*Linum usitatissimum* L.) genome

**DOI:** 10.1186/s13007-015-0062-x

**Published:** 2015-03-14

**Authors:** Leonardo Galindo-González, David Pinzón-Latorre, Erik A Bergen, Dustin C Jensen, Michael K Deyholos

**Affiliations:** Department of Biological Sciences, University of Alberta, Edmonton, AB Canada T6G 2E9; Department of Computing Sciences, Kings University College, Edmonton, AB Canada T6B 2H3; IK Barber School of Arts & Sciences, University of British Columbia, Okanagan campus, Kelowna, BC Canada V1V 1 V7

**Keywords:** *Linum usitatissimum*, Reverse genetics, Ion Torrent, EMS

## Abstract

**Background:**

Detection of induced mutations is valuable for inferring gene function and for developing novel germplasm for crop improvement. Many reverse genetics approaches have been developed to identify mutations in genes of interest within a mutagenized population, including some approaches that rely on next-generation sequencing (e.g. exome capture, whole genome resequencing). As an alternative to these genome or exome-scale methods, we sought to develop a scalable and efficient method for detection of induced mutations that could be applied to a small number of target genes, using Ion Torrent technology. We developed this method in flax (*Linum usitatissimum*), to demonstrate its utility in a crop species.

**Results:**

We used an amplicon-based approach in which DNA samples from an ethyl methanesulfonate (EMS)-mutagenized population were pooled and used as template in PCR reactions to amplify a region of each gene of interest. Barcodes were incorporated during PCR, and the pooled amplicons were sequenced using an Ion Torrent PGM. A pilot experiment with known SNPs showed that they could be detected at a frequency > 0.3% within the pools. We then selected eight genes for which we wanted to discover novel mutations, and applied our approach to screen 768 individuals from the EMS population, using either the Ion 314 or Ion 316 chips. Out of 29 potential mutations identified after processing the NGS reads, 16 mutations were confirmed using Sanger sequencing.

**Conclusions:**

The methodology presented here demonstrates the utility of Ion Torrent technology in detecting mutation variants in specific genome regions for large populations of a species such as flax. The methodology could be scaled-up to test >100 genes using the higher capacity chips now available from Ion Torrent.

**Electronic supplementary material:**

The online version of this article (doi:10.1186/s13007-015-0062-x) contains supplementary material, which is available to authorized users.

## Background

Flax (*Linum usitatissimum* L.) is cultivated as a source of either oil or fiber, both of which have distinct properties that make flax a valuable crop. The oil of flax seeds (i.e. linseed) is rich in polyunsaturated fatty acids including alpha-linolenic acid, which has purported health benefits and is also useful as a drying oil in manufacture of resins, finishes, and flooring. The stem phloem fibers (i.e. bast fibers) of flax are remarkably long and strong and are used for textiles and increasingly as substitutes for fiberglass in composite materials. The commercial potential of flax, as well as interesting aspects of its biology (including well-documented phenotypic and genomic plasticity of some accessions [1]), have led to an increase in research activity in this species, highlighted by the release of an assembly of its whole genome sequence [2]. To accelerate the development of novel germplasm and to better exploit the available DNA sequence resources for flax, we sought to develop a mutant population and a reverse genetics platform for this crop.

Mutations can be induced by treating individuals with physical, biological or chemical mutagens [3]. Ethyl methanesulfonate (EMS) is widely used for inducing point mutations in plants [4-12], and results mostly in G/C to A/T transitions [4] that show a nearly random distribution throughout the genome. While one study showed that the frequency of EMS-induced mutations was estimated at about 1 mutation/300 kb screened [4], the density of mutations can vary for different plants and treatments [12]. Therefore the frequency of Single Nucleotide Variants (SNVs) for sequence length becomes an important factor in the probability of finding a phenotypic effect.

Two main approaches have been developed to relate genotype to phenotype in mutated populations. Forward genetics aims to evaluate the phenotype of hundreds or thousands of individuals to find abnormalities in characteristics like growth or development. Once a phenotypically abnormal individual is identified, map-based cloning or other molecular analyses must be used to identify the DNA sequence that was altered by mutation [13]. In reverse genetics, researchers start with a known DNA sequence of interest, and try to determine the effects of a mutation on the phenotype of the organism [3]. One advantage of reverse genetics is that it overcomes some of the limitations of forward genetics that are caused by functional redundancy [13]. In reverse genetics, mutations in a gene of interest can be obtained even in absence of a clear phenotypic effect, and therefore mutants of related genes can be combined to determine the impact of simultaneous loss-of-function or alteration of two or more genes.

Both forward and reverse genetics require researchers to screen a large number of individuals for the mutation of interest. Several methods have been developed to screen for mutations in a gene of interest within hundreds or thousands of individuals in parallel. TILLING (Targeting Induced Local Lesions in Genomes) was devised as one such methodology. In TILLING, the gene of interest is amplified by PCR of pools of DNA from members of an EMS-mutagenized population. Polymorphisms in the PCR amplicons are detected using denaturing high-performance liquid chromatography (DHPLC), or using a CEL I nuclease preparation, which cleaves the heteroduplexes that form between mutant and non-mutant DNA within the amplicons from the pooled DNA; the activity of the nuclease is then detected by gel electrophoresis [14,15]. TILLING has been used in diverse species including Arabidopsis [4], rice [8], soybean [6], sorghum [9] and tomato [10]. Other alternatives to CEL I- based TILLING have also been described including high resolution DNA melting and conformation sensitive capillary electrophoresis [5,12,16]. However, with the advent of Next Generation Sequencing (NGS) technologies [17-19], the possibilities to improve the efficiency of reverse-genetic screening have increased. NGS provides direct information about the mutated sequence and does not require formation of heteroduplexes. While the cost of sequence is still too high to allow for whole-genome sequencing of every individual in a mutant population of a species such as flax with a genome size of 373 Mb [2], the cost per-reaction may be reduced by incorporation of specific tags or barcodes in the primer sequences, allowing pooling of many samples in a single sequencing run by targeting specific regions of interest. An early approach using NGS to detect EMS mutations on tomato with GS FLX sequencing allowed screening of over 15000 plants [20]; GS FLX has also been used in the evaluation of Tef (*Eragrostis tef*) to examine genes related to lodging resistance [11]. Additional studies have used Illumina technology to perform TILLING by sequencing in rice and wheat [21], and in tobacco [22].

Here we present the first study using an Ion Torrent Personal Genome Machine (PGM) to discover single nucleotide variants (SNVs) or rare variants (these two terms – along with “mutation” - are used interchangeably throughout the text) in an EMS mutant population of an elite linseed variety of flax. The Ion Torrent has one of the lowest instrument and per-run costs of the major NGS platforms [23,24], and its sequencing output is on a scale consistent with the expected requirements of this application. Ion Torrent works with chips bearing millions of microwells with transistor sensors that allow detection of changes in current produced by the release of hydrogen once new nucleotides are incorporated to the clonally amplified DNA strands attached to each one of the beads residing in each well [25]. Massive parallelism can be achieved with this technology and the sequencing capacity limit depends on the number of sensors in the array. During the development of the technology the increases in sequencing throughput have been achieved by growth of the chip size, closer packing of features (e.g. wells) and shrinking of features. In this way for example a 5.2 fold increase in sensor count has been achieved when moving from a 314 chip to a 316 chip [25].

We demonstrate the utility of this approach by identifying SNVs in eight genes of interest, after performing a pilot experiment in three genomic regions with known SNVs to validate the methodology. Our methodology allows identification of putative variants on the target genes and can be scaled up in the number of genes and individuals to screen large populations.

## Results

We conducted three experiments to develop an Ion Torrent-based method for discovery of single nucleotide variants (SNVs) in flax: (i) a pilot experiment with combinations of known SNVs (using an Ion 314 chip); (ii) a proof of concept experiment with a mutagenized population of flax (also using an Ion 314 chip); and (iii) a scale-up experiment using the higher capacity, Ion 316 chip.

### Experiment I: Pilot

To evaluate our ability to detect known variants in selected regions of DNA, we used DNA from two non-mutagenized cultivars of linseed flax: CDC Bethune and Macbeth [26]. We designed primers (Additional file 1) encompassing SNVs that had been previously identified in a comparison of CDC Bethune and Macbeth DNA sequences [27] and designated these regions as S20, S411 and S900 using their scaffold of origin (e.g. S20 = scaffold 20 of the published genome assembly [2]). We mixed DNA from CDC Bethune with DNA from Macbeth to simulate a total of 28 pools from either 64 or 96 individuals, in which one individual in the pool was polymorphic (i.e. carried a SNV not present in any other member of the pool). As a negative control, we also constructed simulated pools that consisted of only DNA from CDC Bethune.

**Figure 1:**
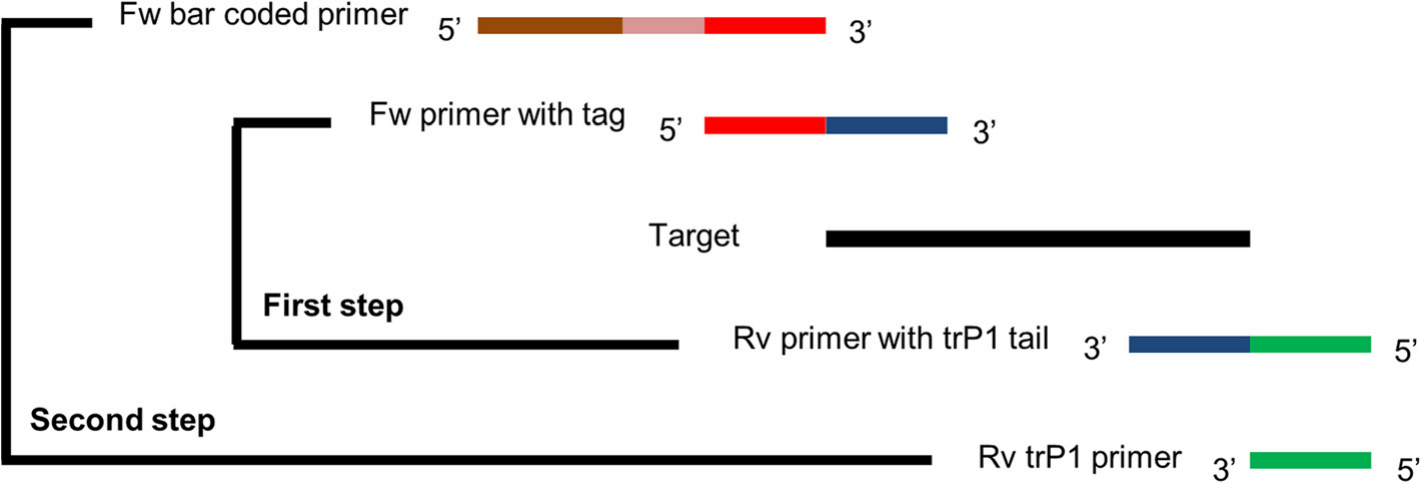
**Two-step PCR strategy adopted for high throughput sequencing.** On the first-step PCR the specific gene section (target) is amplified with a forward primer (blue) bearing a universal tag (red), and a reverse primer (blue) carrying a trP1 adapter (green). For the second-step PCR the amplicons of the first step are amplified with a reverse primer that matches the trP1 adapter (green) and a forward bar-coded (pink) primer (brown) for each desired pool of individuals and genes.

We amplified the three target regions using a two-step PCR (Figure 1 and Additional file 2A-B). The two-step PCR was used because it allowed us to incorporate specific barcodes for each pool (Additional file 3). After the second PCR step, we gel-purified the amplification products to eliminate primer dimers, which could otherwise be preferentially amplified during subsequent emulsion PCR. Gel-purified DNA was diluted to 1 ng μL^−1^, and pooled before diluting all mixed products to 26 pM. This pooled sample was diluted one time to obtain a second pool of half the concentration (13 pM), which was used to perform a second emulsion PCR. We measured the percent of templated Ion Sphere particles (ISPs), as 37.3% for the 26 pM sample and 27.6% for the 13 pM sample. The latter sample was selected for sequencing since the template ISPs fell in the acceptable range of 10 to 30% [28]. The loading of the 26 pM sample was deemed too high for sequencing.

**Figure 2:**
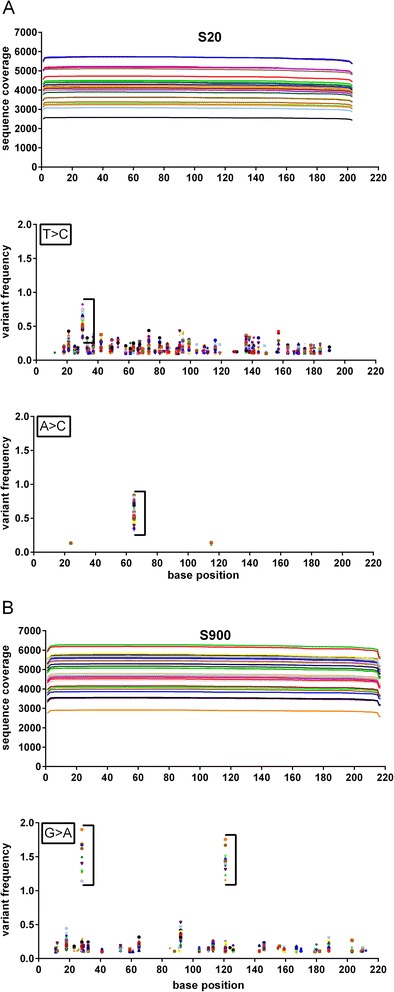
**Sequence coverage and frequency of variants in gene sections of the pilot experiment.** DNA from the cultivar Macbeth was diluted (1:64 or 1:96) in CDC Bethune DNA in several simulated pools as described in methods. Each line in the sequence coverage graphs represents one of 28 pools. The frequencies of the variants are plotted against the position in the respective reference sequence section. Each symbol in the frequency graphs represents a frequency of at least 0.1% for each of the 28 pools in each position. **A**. S20, **B**. S900; no graphs for S411 are shown since no variants were detected in that sequence.

A total of 119.38 Mbp of sequence were obtained which represented 678,532 reads after filtering for polyclonal, dimer and low quality sequences (Table 1). While the modal read length for tested genes was > 200 bp, the mean read size was 176 bp due to a large number of reads in the 50 bp range. These short reads were comprised of incomplete sequence extensions, and sequence artifacts that were filtered out during the subsequent mapping step, leaving 47.35% of all reads to be mapped to the 28 pools (Tables 1 and 2), in each one of the three genomic regions evaluated (Additional file 4). Average read coverage was 4,103, 2,392 and 4,794 for sequences S20, S411 and S900 respectively (Table 2). While coverage did not seem to vary along the sequence, the coverage between pools did vary (Figure 2).

**Table 1:** **Sequencing statistics of the three experiments performed**

**Experiment/replicate**	**Chip type**	**Percentage of wells with beads in chip**	**Total number of bases (Mbp)** ^**1**^	**Total number of reads** ^**1**^	**Percentage of mapped reads to all genes in experiment**	**Mean read length (bp)**
Pilot	314	74%	119.38	678,532	47.35	176
Proof of concept-1	314	79%	71.80	459,888	60.31	156
Proof of concept-2	314	76%	85.40	543,659	63.02	157
Scale up	316	74%	649.00	3,403,220	92.04	190

^1^After filtering polyclonal wells, test fragments, adapter dimers sequences and low quality reads.

**Table 2:** **Read statistics of the three experiments performed**

**Experiment/replicate**	**Scaffold ID/gene ID**	**Gene name/annotation** ^**1**^	**Number of reads mapped in all 28 pools**	**Percentage of mapped reads in all 28 pools** ^**2**^	**Average read coverage per pool**
Pilot	S20	N/A	115,998	17.09	4,103.69
	S411	N/A	67,971	10.02	2,392.08
	S900	N/A	137,314	20.23	4,794.04
Proof of concept-1	Lus10004720	*LuPME10*	91,755	19.95	2,975.50
	G25305^*^	*LuPME73*	94,242	20.49	2,942.75
	Lus10031470	*LuPME79*	38,154	8.30	1,059.81
	Lus10043035	*LuPME105*	53,216	11.57	1,621.20
Proof of concept-2	Lus10004720	*LuPME10*	87,889	16.17	2,815.02
	G25305^*^	*LuPME73*	143,832	26.46	4,404.28
	Lus10031470	*LuPME79*	47,620	8.76	1,255.62
	Lus10043035	*LuPME105*	63,282	11.64	1,941.29
Scale up	Lus10016751	ALS-1	1,443,997	42.43	44,199.34
	Lus10029955	ALS-2	424,425	12.47	13,027.62
	G24175^*^	CLE	534,255	15.70	16,146.73
	Lus10017825	UGT	729,799	21.44	20,472.63

^*^Gene Id correspond to first draft assembly of flax (unpublished).

^1^PME = Pectinmethylesterase, ALS = acetolactate synthase, CLE = cyclic peptide, UGT = glucoronosyl/glucosyl transferase.

^2^Percentage from total number of reads in Table 1.

Analysis of mapped reads from the simulated pools identified expected SNVs in two of the three targeted loci. For S20, a SNV was identified in base 65 (A > C) (Figure 2A). For S900 (Figure 2B), a SNV was identified in base 28 (G > A), but the expected SNV at position 120 (C > T) of S411 that was previously reported was not found in any of the pools. However, novel SNVs (i.e. polymorphisms between Macbeth and CDC Bethune that had not been previously reported) were found in two of the targeted loci: we discovered an additional SNV at position 30 (T > C) in S20, and an additional SNV at position 121 (G > A) in S900 (Figure 2). All of these observations were confirmed by Sanger sequencing of targeted loci (Additional file 5).

### Discovery of EMS-induced mutations in PME genes

Having demonstrated in the pilot experiment that we could detect known SNVs within simulated pools of DNA, we next attempted to discover novel SNVs within pools of DNA obtained from an actual mutagenized population (proof of concept). We used 10 ng of DNA from each of 768 individuals and pooled the DNA as explained in materials and methods. Because of the way our experiment was designed, each one of the 768 individual DNA samples was present in three pools; when a SNV was found in three intersecting pools we could pinpoint the sample of origin. We targeted four genes of the pectin methylesterase (PME) family for discovery of SNVs (*LuPME10*, *LuPME73*, *LuPME79*, *LuPME105*, Table 2). These genes were selected because they are relevant to ongoing cell wall research in our laboratory [29]. To minimize the amplification of primer dimers, we tested the PME primers (Additional file 1), under a range of annealing temperatures and found that the optimal temperature range for the touch-up first-step PCR was 56-66°C (this was higher than the annealing temperature range 50-60°C in the pilot experiment), and the optimal second-step PCR annealing temperature was 68°C. This highlighted the importance of empirically testing PCR conditions for any new set of primers. Amplicons were analyzed and purified on agarose electrophoretic gels, eluted, and quantified (as in the pilot experiment) before Ion Torrent sequencing.

For sequencing, we diluted the pooled PME amplicon DNA to 13 pM. This DNA was sequenced in two replicate runs (to test for consistency). The percent of template ISPs for the two replicates was 23.87% and 20.13%, which made both samples suitable for sequencing. A total of 71.8 Mbp and 85.4 Mbp were obtained with an average read length of 156 bp and 157 bp for the two technical replicates (Table 1). The total number of usable reads after performing filtering of polyclonal, low quality reads and primer dimers were 459,888 and 543,659. However, we found that that even after read filtering, there remained a fraction of short reads in the 50 bp range, which presumably represented primer dimers and incomplete products (Additional file 6).

When comparing to the pilot experiment the percentage of mappable reads increased to over 60% for both replicates (Table 1), but the average read coverage per pool in each of the evaluated genes was proportionally lower than in the pilot since reads in this case were distributed among four genes (Table 2). Furthermore, the proportion of reads mapped was not equally distributed among the four genes in any of the pools. When using the mapped reads in all pools to calculate the coefficient of variation (CV) for each gene and replicate, variability was evident among genes (Additional file 7), but the variation was constant among the two replicates for each gene.

**Figure 3:**
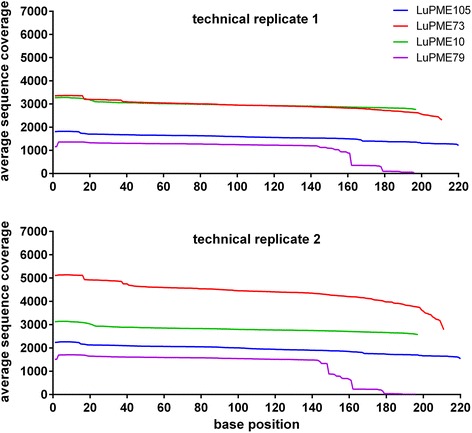
**Coverage of four PME genes in two technical replicates.** The average sequence coverage from 28 pools in each one of the base positions for the four PME genes is shown.

The coverage per position for each gene was high throughout the sequence, with the exception of *LuPME79*, where a drastic decrease in coverage was observed after position 162 of the reads (Figure 3). Analysis of the sequence with Mfold [30] (not shown), did not predict a secondary structure that would explain this apparent hard stop in sequencing. Additionally, GC content of *LuPME79* (57.14%) was similar to *LuPME73* (57.34%), so a bias in GC content could not explain this difference either.

Based on our experience in the pilot experiment, we selected a minimum coverage per position of 500×, with a frequency of at least 0.5% in three intersecting pools, for defining putative mutations. When only two intersecting pools were found with the expected minimum frequency, all individuals from the intersection where sequenced. There was consistency between replicate runs for most SNVs but some of the SNVs were detected by complementary intersecting pools between both replicates. There was no correlation of false positives with the technically consistent SNVs or the ones found by complementarity.

**Figure 4:**
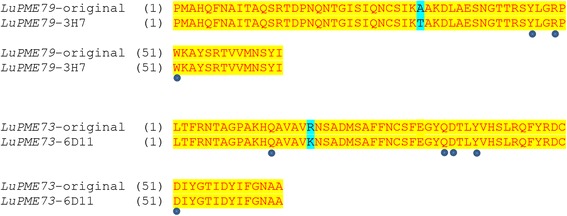
**Alignment of amino acid sections from individuals bearing non-synonymous mutations (Table** 3 **) to the original non-mutated sequences.** Gene IDs are followed by an identifier given to the sequenced individuals. Circles below the alignment indicate enzyme active sites. Blue background indicates the amino acid change.

In our analysis of four targeted PMEs amplified from 768 individuals, we found a total of 13 putative SNVs. Sanger sequencing on the original DNA from the pooled individuals confirmed only five of the 13 putative SNVs (Table 3). When the sequenced sections from the original and mutated individuals were translated, it was found that neither of the two non-synonymous changes found was within the predicted enzyme active sites [31] (Figure 4). Nevertheless, the methodology proved useful for finding mutations in pooled mutated populations when testing several genes at the same time.

**Table 3:** **SNVs found in four PME genes**

**Gene**	**Base No.**	**Change**	**Sanger confirmation**	**Nucleotide substitution**	**Amino acid substitution**
*LuPME79*	33	G > A	No	N/A	N/A
*LuPME79*	96	G > A	Yes	Heterozygous	Non-synonymous
*LuPME73*	25	G > A	No	N/A	N/A
*LuPME73*	54	G > A	Yes	Heterozygous	Non-synonymous
*LuPME73*	81	T > A*	No	N/A	N/A
*LuPME73*	88	C > T	Yes	Heterozygous	Synonymous
*LuPME73*	97	A > G*	No	N/A	N/A
*LuPME73*	139	C > T	Yes	Homozygous	Synonymous
*LuPME73*	189	C > T	No	N/A	N/A
*LuPME10*	154	C > T	Yes	Homozygous	Synonymous
*LuPME105*	34	A > G*	N/A	N/A	N/A
*LuPME105*	57	A > G*	No	N/A	N/A
*LuPME105*	115	G > A	No	N/A	N/A

N/A – sequence could not be obtained by Sanger.

*Mutations not expected by EMS, but discovered using the technique.

### Increased read depth for discovery of EMS-induced mutations

Because the previous experiment showed a large variation in mapped reads between genes and read depth among pools in each gene (Table 2 and Additional file 7) and less than half of the predicted SNVs could be confirmed by Sanger sequencing, we decided to increase read depth by switching from Ion 314 chips to the higher capacity Ion 316 chips (scale up). We used four genes related to flax metabolism (Additional file 1 and Table 2). These genes are related to characteristics related to bitter taste in flax (cyclic peptides), targeting of group 2 herbicides (acetolactate syntases), or important as major components of cell wall formation (glucoronosyl/glucosyl transferases). We selected regions in these genes based on previous studies showing critical sections and/or amino acids for the function of these proteins [32-35].

We tested again two dilutions at 13 and 26 pM to assess which of these would give a better percentage of template ISPs. We obtained 12.15 and 17.35% of templated ISPs respectively and sequenced only the latter sample, which had the highest percent loading. A total of 649 Mbp were obtained with an average read length of 190 bp (Table 1). The total number of usable reads after filtering was 3,403,220 which was an approximately 5-fold increase from the 314 chips used in the first two experiments. Although the coverage of pools among genes fell slightly towards the end of the sequences (result not shown), the average coverage for the four evaluated genes was 10 times higher than in the previous experiment (Table 2), and therefore the depth was sufficient to assess variants in any position throughout pools and genes.

Using similar parameters as for the PMEs, we found a total of 16 putative SNVs from which 11 were confirmed by Sanger sequencing (Table 4). From these, two were found to be homozygous. One of the heterozygous mutations resulted in the generation of a stop codon.

**Table 4:** **SNVs found in four genes of interest**

**Gene**	**Base No.**	**Change**	**Sanger confirmation**	**Nucleotide substitution**	**Amino acid substitution**	**Sanger confirmation on M**_**4**_	**Nucleotide substitution in progeny** ^**1**^	**Script confirmation** ^**2**^
ALS-1	119	C > T	Yes	Heterozygous	A/V	Yes	2 homozygous	Yes^++^
ALS-1	140	C > T	No	N/A	P/L	N/A	N/A	No
CLE	89	G > A	Yes	Heterozygous	G/D	Yes	3 heterozygous	Yes^+++^
CLE	94	G > A	Yes	Heterozygous	E/K	Yes	1 homozygous, 1 heterozygous	No
CLE	134	G > A	Yes	Heterozygous	R/H	Yes	1 homozygous	Yes^+++^
ALS-2	26	G > A	No	N/A	G/E	N/A	N/A	No
ALS-2	43	G > A	Yes	Heterozygous	E/K	Yes	2 homozygous	No
ALS-2	100	G > A	No	N/A	E/K	N/A	N/A	No
ALS-2	161	C > T	Yes	Heterozygous	A/V	Yes	2 homozygous	Yes^+++^
ALS-2	161	C > T	Yes	Homozygous	A/V	Yes	2 homozygous	Yes^+^
UGT	27	C > T	No	N/A	P/S	N/A	N/A	No
UGT	33	C > T	No	N/A	H/Y	N/A	N/A	No
UGT	81	C > T	Yes	Homozygous	L/F	Yes	3 homozygous	Yes^+++^
UGT	99	G > A*	Yes	Heterozygous	E/STOP	Yes	3 heterozygous	N/A
UGT	99	G > A*	Yes	Heterozygous	E/STOP	Yes	2 heterozygous	N/A
UGT	184	G > A	Yes	Heterozygous	G/E	Yes	1 homozygous, 1heterozygous	No

^*^Mutation was found by looking at intersecting pools with frequencies below the set threshold.

^1^Six individuals from progeny examined per mutation.

^2^The frequencies of the variants were used to run a Phyton script which automatically detects the source individual bearing the mutation (Additional file 8). Parameters used in the script were: 2 lower SD cutoff, 10 upper SD cutoff, 1 min. pools. N/A rows were not picked by the script since they were found by a different methodology. The confirmed points by the script had confidence intervals of: ^+^75%, ^++^85% and ^+++^99%.

Mutations on position 161 on ALS-2 and 99 on UGT were found in two different individuals.

We tested the heritability of the SNVs discovered in ALS1, ALS2, UGT and CLE by Sanger sequencing of the progeny of plants in which the mutations were initially identified. The presence of the mutation was confirmed in the progeny of all of the lines (Table 4).

## Discussion

### Ion Torrent technology in SNV detection

The advent of next-generation sequencing technologies has opened new doors for genomic-scale analyses [18,19]. Among common sequencing platforms IonTorrent offers potential advantages including low instrument cost, low cost per base, and fast output (up to 333 Mbp/h) [23,36]. Ion Torrent has also been reported to be superior for variant calling than Illumina [23], although other studies report similar or slightly higher sensitivity for MiSeq [37]. While Ion Torrent has a high rate of indels caused by homopolymeric runs, and long-range sequence quality can be lower than that of other instruments [23,36,38,39], this is not a problem for calling SNVs with high read depth.

We used Ion 314 and Ion 316 chips and were able to reach reads in the 200 bp range with a total sequence throughput that guaranteed high depth. The size reached by these reads facilitated the evaluation of critical gene regions without the need for post-sequencing assembly. Likewise, we were able to achieve a high-throughput in the number of samples analyzed for several genes. A similar approach used Ion Torrent to map mutations in mice, but examined a large number of regions in a few samples [40].

In the pilot experiment, we detected the expected mutations and additional unreported changes in the tested genomic regions (Figure 2 and Additional file 5). Our experiment with PMEs had a larger variability in read depth among pools and genes (Additional file 7), which may have had an influence on the number of false positives. When we increased our read depth by using the larger Ion 316 chip, the number of false positives decreased significantly.

Several variables were optimized during our experiment. Since sample pooling was used, high-quality DNA in equal amounts was needed to increase the probability of detecting one mutated individual among the population. We standardized a high-throughput CTAB protocol yielding high quality DNA and further quantified the samples by fluorescence to add equal amounts of DNA from each individual in each pool. Additionally, the first step PCR required addition of different PCR additives (ethylene glycol and DMSO), which decreased the formation of secondary structures since preliminary tests showed that a standard PCR resulted in a high proportion of primer dimers (or secondary structures) due to the length of the primers; nevertheless some residual dimers were still unavoidable (Additional file 2). A PCR cleanup did not suffice to get rid of all dimers, which were still carried over to the emulsion PCR, resulting in a preferential amplification of these smaller products in preliminary runs (result not shown). Eluting the specific products from agarose gels improved the detection of the larger specific products but still with some residual carryover. A better primer removal method like a solid-phase reversible immobilization (SPRI) technology was suggested for the process of PCR cleanup for Illumina [41], and could also be used in future experiments.

Differences in read coverage were detected in our different experiments, but this is not uncommon in NGS technologies [21]. As a general trend we found that shorter amplicons resulted in higher average coverage over all positions in all pools for the evaluated gene sections of the three experiments. This can be related especially to emulsion PCR, since this constitutes a step where all genes are mixed and there can be a preferential amplification of shorter amplicons. Factors like shorter denaturation times and faster extension on smaller products may lead to this preferential amplification [42].

However, the exact same relationship was not found for the PMEs (Figure 3). The two PME amplicons corresponding to *LuPME79* and *LuPME105* had a lower average coverage, and while the later does correspond to the larger amplicon of this gene set, the former is the smallest. Therefore different factors may have had an influence on the variability in read count that we encountered. When we calculated GC content it was seen that *LuPME105* had the lowest GC content (43%) of the four PME sections evaluated. Ion Torrent read coverage has been shown to decrease upon high or low GC content or under different levels of genome complexity [23,25,43]. Likewise, gel extraction has been shown also to have a bias for recovery of GC-rich double stranded templates that have higher affinity for kit columns, than AT-rich amplicons which become single stranded upon agarose melting conditions [41].

Neither length, nor GC, seemed correlated with the lower coverage of *LuPME79*, however, the primers from *LuPME79* and *LuPME105* (Additional file 1) had a lower value of Gibbs energy – ΔG - (−11.32 and −10.24 kcal mol^−1^ respectively) compared with the primers of *LuPME10* and *LuPME73* (−5.19 and −8.48 kcal mol^−1^). Since a lower ΔG favors the formation of secondary structures, this could have had an effect of the PCR resulting in a differential amount of amplicons before pooling. Unforeseen changes like EMS mutations in priming sites can also contribute to differential amplification among samples. We also encountered a drop in coverage after position 162 in *LuPME79*; while we could not detect any evident secondary structure after the hard stop in the sequence reads, 16 out of the 20 previous nucleotides before the read coverage fall are G or C and this could be related to the formation of a secondary structure that impairs the sequencing polymerase from continuing.

Length and GC content of the target locus are not entirely under control of the researcher. However, other factors can be better controlled to achieve near-homogeneous coverage when pooling samples for analysis. For example, an accurate quantification of the PCR products after the first round of PCR by comparison to a standard [44], would decrease biases in amounts of amplicons before pooling. Although previous studies have shown that non-normalized samples are suitable to detect high-frequency variants [44], our study comprised the detection of mutations in pooled samples, where a homozygous mutation could theoretically have a low frequency: aprox. 1% for a 1 in 96 dilution, and of 0.5% for an heterozygous allele. Additionally, very small amounts of DNA (10 ng) were used in the pools. Since differences in the amount of starting DNA can also result in differential amplification [42], it is important to guarantee close to equimolar amounts of starting DNA to avoid losing a variant due to a PCR deficiency.

Overall, the coverage on the 11 different gene regions that were tested allowed for the detection of SNVs in regions of up to 200 bp. Although coverage varied slightly between some pools and genes, coverage along the length of amplicons was generally even but dropped only towards the ends of the sequences (Figure 3), which is common of sequencing-by-synthesis technologies [25]. With further optimization, the Ion 314 chips could easily accommodate the evaluation of 16 amplicons at an average of more than 500× coverage per pool under the 28-pool scheme we utilized. Theoretically, for the Ion 316 chip, 160 amplicons could be evaluated under the same conditions, but adjusting the technical parameters under the current technology to guarantee little variability from pooling to sequencing becomes harder unless a similar system to the Ampliseq, used for human genes [25], can be rapidly implemented for plants.

Another factor that came into consideration was how to decrease the level of false positives in data. Whereas it is has been reported that the level of false positives in Ion Torrent data is larger than Illumina, it has also been shown that Ion Torrent can detect more true positives given enough coverage [23]. From our data it was evident that the increase in depth upon using the Ion 316 chip was concomitant with a decrease in false positives (compare Tables 3 and 4), showing that read depth is key for separating real mutations from noise in SNV studies [21], and in studies where allele frequency needs to be resolved [45]. While our proof of concept and scale experiments differed in the amplicons used, read mapping statistics (Tables 1 and 2) demonstrated that the experiment with the 316 chip had an increase in the number of reads by over 5 fold when compared to the 314 chip. Since other factors like GC content and small differences in amplicon size are still difficult to control for and do not have a clear correlation always with read depth, we believe that the technical increase on read number by selecting a higher capacity chip is the key factor in obtaining more rare variants. Unfortunately, most second generation NGS technologies still present high error rates (see below). While we tried to control for equimolar amounts of DNA when pooling samples, PCR steps result in uncontrollable dilutions of some samples before sequencing which will result in the loss of some SNVs among noise. Improvements to eliminate such technical variability will increase our ability for SNV detection.

There are additional elements to have into account. For example, it was noticed that the background frequency of base substitution varied between the type of substitutions and among genes. While an A > C change had little or no background over 0.1% for the S20 region (see Figure 2), a T > C change in the same region and a G > A in the S900 region had larger substitution noise. A differential rate of substitution has been linked to the PGM from Ion Torrent upon studying bacteria, with G > A and T > C transitions presenting the higher rates of substitution with the Ion OneTouch 200 template kit [39].

Interestingly while homopolymer errors are the most common error type from this technology [23,36,39], an study with bacteria found that substitutions have the highest variation frequencies, with standard deviations ranging from 26%-56% of the mean substitution rate [39]. This has implications in the detection of rare variants (including false negatives) which may come up at lower frequencies as we detected in our study due to sample dilution in pools. For example, a 0.3% frequency was found for S20 variants in the pilot experiment (Figure 2), and since some random error can reach this frequency, this can lower our detection ability. While there were a few false positives embedded into homopolymeric tracts which constitute the bigger source of error of Ion Torrent technology [39], no specific position or sequence-specific bias in the SNVs that were not confirmed by sanger could be inferred to make any generalization.

Compared to Roche/454 technologies where mutagenized populations are used to discover rare variants [11,20] the Ion Torrent technology offers a higher read depth in short times, which results in a higher probability to find the mutated bases. While one of the main advantages of 454 sequencing over other technologies was their read length (>400 bp), Ion Torrent is quickly catching up to offer similar read lengths of high quality [25]. Our study achieved similar throughput (314 chips) in the number of reads obtained as the aforementioned 454 studies, but the time required for a run on an Ion Torrent PGM is just over 2 hours while the most basic 454 sequencers use at least 10 hours and with prices of equipment and cost per base which are above the ones of Ion Torrent [36,46]. Furthermore, higher throughput was achieved (5-fold) when changing to a 316 chip without a change in runtime while for 454 technology the use of higher-end sequencers may require up to 23 hours for a run [46]. Finally, the rate of false positives seems to be on a similar range for these two technologies.

Our methodology was based on a previous Illumina study [21] and therefore some conclusions can be drawn by comparing the two. It was clear that because of the similar methodology similar results were obtained in several fields. For example low coverage resulted in increased noise which impaired SNV detection in both studies. The throughput of Illumina is generally higher resulting in detection of many more mutations. However, this is achieved in longer runtimes (days) and with more expensive equipment, although cost per base is lower on Illumina [36,37,46]. Because of the higher error rates of the Ion Torrent technology the amount of false positives is usually higher than on Illumina [23].

## Conclusion

We have demonstrated that the Ion Torrent can be used in a scalable, amplicon-based approach for efficient discovery of mutations in a small number of genes. The efficiency of the method is limited by the rate of false positives, which may be decreased by higher read-depth and further technical optimization. Ion Torrent technology has been demonstrated to introduce biases at errors at specific steps during sequencing [43], as have other sequencing technologies, especially when PCR step is used in sample preparation [47]. Nevertheless, the Ion Torrent PGM platform detected rare variants with as low as 0.3% frequency per pool according to our results, which is above the substitution error of 0.1% calculated for the technology [25], and we showed that 768 individuals could be easily pooled per run. The Ion Torrent is one of the first technologies that does need optical systems to detect nucleotide incorporation, and does not use modified nucleotides [25,48]; in addition it has a good combination of throughput, cost and time saving compared to other systems [23,36,44,49]. The use of chips with larger capacity [25], will allow increasing both the number of genes and/or pooled samples. Additionally, the availability of 400 bp kits now allows exploring larger regions of interest without the need of using paired ends.

## Methods

### Plant material

Seeds of *Linum usitatissimum* L. (var. CDC Bethune), an elite linseed cultivar, were obtained from Gordon Rowland (Crop Development Center, Saskatoon, SK). Seeds were soaked in 5 volumes (liquid volume/seed volume) of 0.5% ethyl methyl sulfonate (EMS) in 25 mM phosphate buffer (pH 7.6) for 4 h at room temperature and then were rinsed with distilled water (three times), and air dried prior to storage. These M_1_ seeds were sown at the University of Alberta farm (Edmonton, AB), and their M_2_ progeny were harvested as individual families. Approximately four seeds from each M_2_ line were sown in rows at Kernen Farm (Saskatoon, SK) in summer 2010. Leaves were harvested and lyophilized for subsequent DNA extraction, and their progeny (i.e. M_3_ families) were harvested from individual plants, then threshed and stored at ambient temperature in envelopes until screening.

### DNA extraction and pooling

DNA extraction was performed for 96 samples at a time using CTAB [50] with some modifications. Lyophilized leaf samples (10–20 mg) were placed in 8-strip 1.2 mL collection tubes (QIAGEN, Hilden, Germany) containing a sterile 3 mm tungsten carbide beads. Tubes were capped and ground for 2 minutes at 25 Hz using a Retsch MM301 mixer mill (Retsch, Haan, Germany). Samples were centrifuged at 1,450 × g for 1 minute. CTAB was prepared with 2% CTAB (w/v), 2% PVP-40, 100 mM Tris-Cl pH 8.0, 25 mM EDTA pH 8.0, 1 M NaCl and 0.5 g L^−1^ of spermidine. The buffer was pre-warmed (60°C) and supplemented with 10 μg mL^−1^ of RNAse A (Sigma-Aldrich, St. Louis, MO, USA), 100 μg mL^−1^ of proteinase K (Fermentas, Waltham, MA, USA) and 5% mercaptoethanol, before adding 500 μL to each sample. The tubes were re-capped with new caps and mixed by inversion (20 times) and incubated at 60°C for 2 hours, mixing the tubes by inversion every 20 minutes. After incubation, samples were centrifuged for 5 minutes at 5,800 × *g* and the supernatants were transferred to new tubes. Five hundred microliters of chloroform : isoamyl (24:1) were added to each sample and re-capped tubes were mixed by inversion (60 times) before centrifugation for 5 minutes at 5,800 × *g*. The supernatant was transferred to new tubes. Chloroform : isoamyl extraction was repeated once again. Three hundred microliters of ice-cold isopropanol were added to the tubes with the supernatant and the samples were mixed by inversion (20 times) before transferring to −20°C for 2 hours. Incubation was followed by centrifugation at 5,800 × g for 15 minutes. Supernatants were decanted to waste and 500 μL of ice cold 95% ethanol were added to the pellets and samples were gently vortexed prior to centrifugation at 5,800 g for 5 minutes. The previous step was repeated with 500 μL of ice cold 70% ethanol. The ethanol was decanted and the samples were air-dried, and resuspended in 125 μL of TE 10:1 and stored at −20°C.

DNA samples were quantified using Picogreen (Invitrogen, Carlsbad, CA, USA) in a Fluorostar BMG plate reader (BMG labtech, Ortemberg, Germany), using a standard curve of flax DNA. Samples were diluted to 10 ng μL^−1^ and 1 μL or each pooled in groups of 64 or 96. According to the formula used by Tsai et al. [21] using 10 ng of DNA with a flax genome of 2C = 0.764 pg [2] would yield 204.5 copies per allele (for each individual) for the 1 in 64 dilution or 136.3 copies per allele for the 1 in 96 dilution. This is higher than the minimum number of recommended copies (40) to avoid absence or fluctuation of copies among individuals [21].

The pools were created using the following methodology: each sample was in an individual well of eight 96-well plates; all individuals from each plate were pooled creating the first eight pools containing 96 individuals each (designated pools A1 to A8). Then all the individuals from the same column in each plate were pooled creating 12 pools of 64 individuals each (designated pools B1 to B12). And finally all individuals from the same row in each plate were pooled creating the last eight pools of 96 individuals each (designated pools C1 to C8). In this way each individual was part of three different pools and therefore a mutation detected in three intersecting pools would allow us to pinpoint the source individual.

### Primer design

The primers were designed using Primer 3 [51] with the following parameters: Two C’s or G’s in the last five nucleotides towards the 3′ end; up to three nucleotides long homopolymers; a delta G lower than −9 kcal/mole, Tm between 59 and 61°C and size between 19 and 21 nucleotides (when conditions were not met parameters were relaxed). For the forward primer the universal primer tag 5′-CAGTCGGGCGTCATCA-3′ was added (designed by Travis Glenn, Univ. of SC, http://www.gvsu.edu/dna/universal-primer-tag-6.htm) and for the primer Rv the adaptor trP1 was added (5′-CCTCTCTATGGGCAGTCGGTGAT–3′) (Additional file 1).

### Pilot experiment

A pilot experiment was performed using known SNVs showing polymorphisms between cultivars CDC Bethune and Macbeth. Since for the pilot experiment we were not trying to discover new mutations we did not have to pool DNA from different individuals as described in the DNA extraction and pooling section; instead Macbeth DNA was diluted 1:64 or 1:96 in Bethune DNA to simulate the presence of an individual with a mutation within the population. Three genomic regions with previously reported SNVs [27], from three flax scaffolds were chosen to test the methodology and were named S20, S411 and S900 (names were derived from the names of the scaffolds that contained them - see Additional file 1).

### PCR amplification and barcoding

A two-step PCR strategy was adopted: the first step used a PCR with two sets of cycles at different temperatures (below) to amplify the target gene and the second step incorporated a barcode oligonucleotide to distinguish different pools (Figure 1). First step PCR was performed on each of 28 sample pools and three genes per pool (pilot experiment), with forward primers bearing a universal tag at their 5′ end (Additional file 1), and reverse primers carrying an additional adaptor tail which binds the ionospheres used in the sequencing step.

For the pilot experiment, first-step amplifications were performed on a template that either included DNA with known SNVs S20, S411 and S900 (Macbeth DNA in 1:64 or 1:96 dilution in CDC Bethune DNA) or homogeneous template without the SNVs (i.e. only CDC Bethune DNA). The PCR products were diluted 1:100 and equal amounts (5 μL) of PCR product from each of the three target regions were mixed in each of 28 pools for barcoding; the changes of Macbeth genic regions SNVs where introduced in all pools for the gene of S20, and in 10 pools for genes S411 and S900. For the second step, a total of 28 PCRs were performed with forward bar-coded primers (Figure 1 and Additional file 2), and a trP1 primer complementary to the tail from the reverse primer of the first-step PCR. The 28 bar codes allowed us to discriminate between the respective DNA pools after sequencing.

First step PCR was carried under the following conditions: 1X PCR buffer, 2 mM Mg, 0.2 mM dNTPs, 0.2 μM of each forward and reverse primers (Additional file 1), 1 M ethylene glycol, 4% dimethyl sulfoxide (DMSO), 10 ng of DNA and 1.25 units of Platinum Taq DNA polymerase (Invitrogen, Burlington, ON, Canada). The PCR protocol included an initial denaturation step at 94°C for 3 minutes, followed by 5 cycles of 94°C for 20 seconds, 50°C for 30 seconds and 72°C for 1 minute. Finally, an additional 25 cycles were done by changing the annealing temperature to 60°C (touch-up), and a final extension step was performed at 72°C for 10 minutes.

The second-step PCR was carried out as follows: 1X PCR buffer, 2 mM Mg, 0.2 mM dNTPs, 0.2 μM forward bar-coded primer and 0.2 μM reverse trP1 primer (Additional files 1 and 3), 1 μL of the DNA amplicon dilution (three genes combined per pool) and 1.25 units of Platinum Taq DNA polymerase (Invitrogen, Burlington, ON, Canada). The PCR protocol included an initial denaturation step at 94°C followed by 30 cycles of 94°C for 30 seconds, 62°C for 30 seconds and 72°C for 1 minute, and a final extension of 72°C for 10 minutes.

### Purification and quantification of PCR products

The products of PCR reactions of 28 pools and 3 genes per pool were run on 1.5% agarose gels. To eliminate primer dimers, a band of the expected size was gel purified using the Wizard SV gel and PCR clean-up system (Promega, Madison, WI, U.S.A). Samples were quantified using a Qubit 2.0 fluorometer using a dsDNA HS assay (Invitrogen, Burlington, ON, Canada). All quantified samples were diluted to 1 ng μL^−1^ and equal amount of the PCR products were combined and were re-measured on the Qubit to confirm that concentration was still 1 ng μL^−1^. The sample was diluted with low Tris EDTA buffer (TE 10:1) to obtain 15.5×10^6^ molecules per microliter (26 pM) which is the recommended concentration for template preparation using Ion Torrent technology.

### Sequencing

All procedures for emulsion PCR and next-generation sequencing were performed with Ion Torrent equipment and Ion Torrent kits under the manufacturer specifications (Life Technologies, Carlsbad, CA, U.S.A.): emulsion PCR was performed with the Ion OneTouch 200 template kit in an Ion OneTouch. Enrichment of template positive Ionospheres (ISPs) was performed with an Ion OneTouch ES (Life Technologies, Carlsbad, CA, U.S.A.). Sequencing of enriched templates bound to ionospheres was done using the Ion PGM 200 sequencing kit in an Ion PGM Sequencer with either 314 or 316 chips. FASTQ files of each barcoded group of sequences were recovered from the Ion Torrent server for further analysis. Reads have been deposited in the Sequence Read Archive (SRA) from NCBI under study accession number: SRP052626.

### Detection of induced mutations in a population of EMS mutagenized flax

Procedures were similar to the pilot experiment, unless stated otherwise. The experimental design was adapted from Tsai et al., 2011 [21]. A total of 28 pools of DNA from distinct individuals (768 lyophilized leaf samples) were created to facilitate detection of mutations as described in DNA extraction and pooling.

A total of eight primer pairs that amplified pectin methylesterases (PMEs) were designed to target conserved regions presumed to be essential for enzymatic function and tertiary structure stability of these genes [29,31]. Preliminary tests showed that four of the primers pairs (Additional file 1), gave stronger products, and these were used for second-step PCR as described above. Separately, 12 primer pairs from three different metabolism-related genes that constitute important breeding traits (cyclic peptides, acetolactate synthase and UDP - glucuronosyl/glucosyl transferases) were also designed and four primer pairs were selected after testing them by PCR (Additional file 1).

### Analysis of Single Nucleotide Variants (SNVs)

Reads obtained from Ion Torrent PGM sequencing were uploaded to the CLC Genomics workbench platform (CLCbio, Aarhus N, Denmark). Reads were mapped to reference sequences previously confirmed by Sanger capillary sequencing of target amplicons (data not shown), using the following parameters: masking mode = no masking, mismatch cost = 2, insertion cost = 3, deletion cost = 3, length fraction = 0.8 and similarity fraction = 0.8, global alignment = no, non-specific match handling = map randomly, output mode = create stand-alone read mapping, create report = yes, collect unmapped reads = yes. Once the reads were mapped to the reference, the mapped reads files were used as input to discover rare variants using quality score with the following parameters: neighborhood radius = 5, maximum gap and mismatch count = 5, minimum neighborhood quality = 15, minimum central quality = 20, ignore non-specific matches = yes, ignore broken pairs = yes, minimum coverage = 100, minimum variant frequency (%) = 0.1 (selected according to a previous study [25]), maximum expected alleles = 4, advanced = no, require presence in both forward and reverse strands = no, filter454/ion homopolymer indels = yes, create track = yes, create annotated table = yes, genetic code = 1 standard. The whole process was automated by creating a CLC workbench workflow. We also generated a Python script to automate mutation identification in the future (Additional file 8).

Frequency tables were created manually using Microsoft Excel per each gene and per each pool after filtering homopolymeric tracts and indel artifacts created by the sequencing technology. Graphs showing the frequency changes by position for the 28 pools in a specific base change (e.g. G to A) were done to detect outliers visually which were indicative of a rare variant. SNV candidates were chosen if the mutation was present in three intersecting pools or in two intersecting pools with high frequency (>0.3% in pilot experiment and >0.5% in remaining experiments). DNAs from individuals (M_3_ generation) with potential mutations were re-sequenced using Sanger sequencing to confirm the analysis performed with Ion Torrent.

## Additional files

Additional file 1:
**Primer sequences and adaptors used for pilot and test studies.**
Additional file 2:
**Two-step PCR from pilot experiment.** A. Products of first step PCR from the pilot experiment. Amplifications were performed with 5 or 10 ng of pooled DNA in the different dilutions that simulated the inclusion of the mutated individual. Gel was run in 1.5% agarose in TAE 1X at 90 V for 40 minutes. Size of marker bands is given in bp. Negative control refers to a PCR with no DNA template. B. Products of second step PCR from the pilot experiment. Amplifications were performed with 1:100 dilutions of the first-step PCR mixed products of the three genes (S20, S411, S900). Gel was run in 1.5% agarose in TAE 1X at 90 V for 60 minutes. Size of marker bands is given in bp. Negative control refers to a PCR with no DNA template. Additional file 3:
**Primer sequences (with barcodes) and adaptors used for second step-pcr.**
Additional file 4:
**Sanger-sequenced fragments of references genes used in the different experiments.**
Additional file 5:
**Alignment of sequenced fragments of the pilot experiment.** The number 1 over the alignment indicates position 1 for reference of mutations found. Additional file 6:
**Ion Sphere Particles (ISPs) and read identification summary.** The data is given for two technical replicates runs (A and B) of PMEs using the Ion Torrent PGM. Additional file 7:
**Average read count and dispersion among the mapped reads for two replicate sequencing runs of PME genes.** Statistics are given for the 28 pools for all mapped reads or for each individual gene. Additional file 8:
**Python script algorithm description.** The methodology, results and discussion using this script are presented.
