# Supplementary material for: Ion Torrent sequencing as a tool for mutation discovery in the flax (Linum usitatissimum L.) genome
Source: Plant Methods. 2015 Mar 14;11:19. doi: 10.1186/s13007-015-0062-x (PMC4363359; doi:10.1186/s13007-015-0062-x)
Supplement: Additional file 2: — Two-step PCR from pilot experiment. A. Products of first step PCR from the pilot experiment. Amplifications were performed with 5 or 10 ng of pooled DNA in the different dilutions that simulated the inclusion of the mutated individual. Gel was run in 1.5% agarose in TAE 1X at 90 V for 40 minutes. Size of marker bands is given in bp. Negative control refers to a PCR with no DNA template. B. Products of second step PCR from the pilot experiment. Amplifications were performed with 1:100 dilutions of the first-step PCR mixed products of the three genes (S20, S411, S900). Gel was run in 1.5% agarose in TAE 1X at 90 V for 60 minutes. Size of marker bands is given in bp. Negative control refers to a PCR with no DNA template. [file 13007_2015_62_MOESM2_ESM.docx]

**Additional file 2.** Two-step PCR from pilot experiment.

A. Products of first step PCR from the pilot experiment. Amplifications were performed with 5 or 10 ng of pooled DNA in the different dilutions that simulated the inclusion of the mutated individual. Gel was run in 1.5% agarose in TAE 1X at 90V for 40 minutes. Size of marker bands is given in bp. Negative control refers to a PCR with no DNA template.

**Samples primer DNA-dilution proportion cultivars**

First - marker

1 - 2 S20 5ng-10ng Macbeth:Bethune 1:96

3 - 4 S20 5ng-10ng Macbeth:Bethune 1:64

5 - 6 S20 5ng-10ng Bethune 100%

7 S20 negative negative

8 - 9 S411 5ng-10ng Macbeth:Bethune 1:96

10 - 11 S411 5ng-10ng Macbeth:Bethune 1:64

12 - 13 S411 5ng-10ng Bethune 100%

14 S411 negative negative

15 - 16 S900 5ng-10ng Macbeth:Bethune 1:96

17 - 18 S900 5ng-10ng Macbeth:Bethune 1:64

19 - 20 S900 5ng-10ng Bethune 100%

21 S900 negative negative


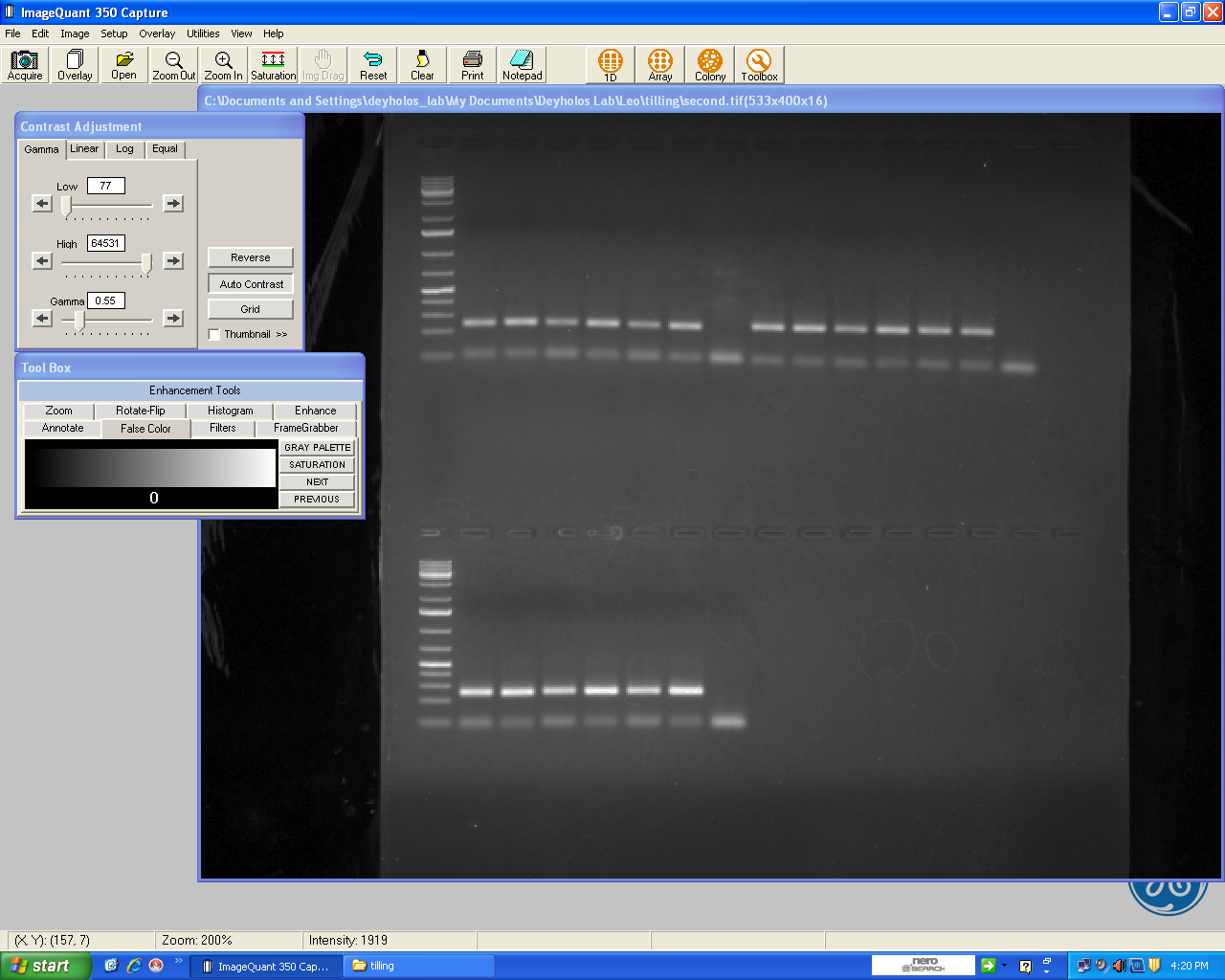


1 2 3 4 5 6 7 8 9 10 11 12 13 14

15 16 17 18 19 20 21

300

500

400

200

B. Products of second step PCR from the pilot experiment. Amplifications were performed with 1:100 dilutions of the first-step PCR mixed products of the three genes (S20, S411, S900). Gel was run in 1.5% agarose in TAE 1X at 90V for 60 minutes. Size of marker bands is given in bp. Negative control refers to a PCR with no DNA template.

**samples tRP1 and barcoded primer**

First - marker

top 1-14 barcode primers 1-14

bottom 1-14 barcode primers 15-28


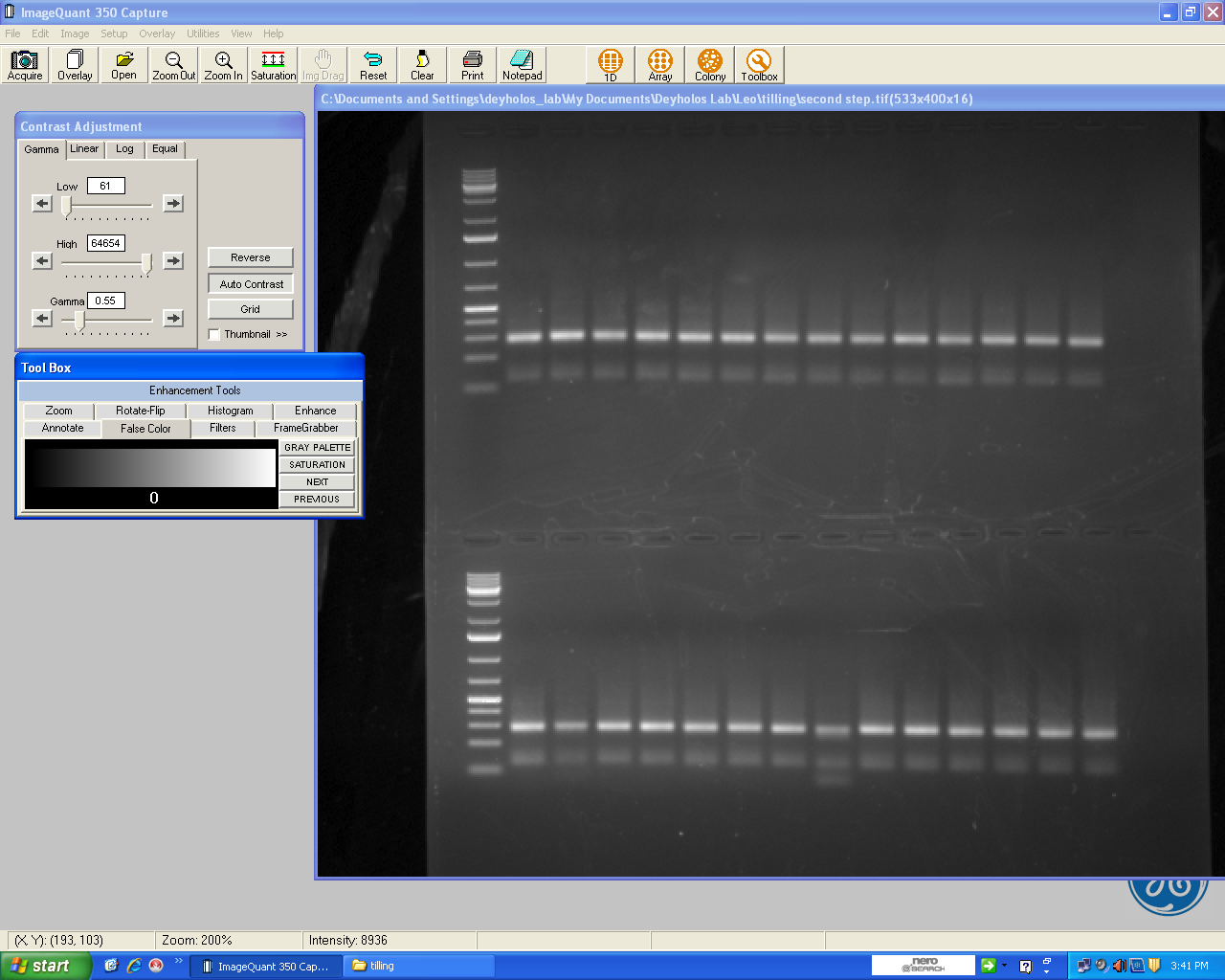


1 2 3 4 5 6 7 8 9 10 11 12 13 14

15 16 17 18 19 20 21 22 23 24 25 26 27 28

300

500

400

200
