# Supplementary material for: Ion Torrent sequencing as a tool for mutation discovery in the flax (Linum usitatissimum L.) genome
Source: Plant Methods. 2015 Mar 14;11:19. doi: 10.1186/s13007-015-0062-x (PMC4363359; doi:10.1186/s13007-015-0062-x)
Supplement: Additional file 4: — Sanger-sequenced fragments of references genes used in the different experiments. [file 13007_2015_62_MOESM4_ESM.docx]

**Additional file 4.** Sanger-sequenced fragments of references genes used in the different experiments.

**Pilot experiment**

>scaffold20_(203bp)-S20

CTCCGTCATGGTATTAGTCATGAATTTACTACTTTTTTCACTGCACATTCCATGACATAATCTGATTCTAACTGCATCGTTTTGTTAGAGTTTCTGGCTCGGATGGATTGTTAAATCATGGCCACTATTTTGGGCTCTCTTCATCAGTTTTGTCCTTGGCACTGCCTATTCAATCAATGTAAGTTCAGTTCGGCTCTATGTTC

>scaffold411_(209bp)-S411

GGAAACATACTGGTCCTCTTCGCTGGTCATAAACGGTACACGGACCGATCCATCATTCAGTAGGTGAAAATCATAGCGTTTAGTTGCCGATGCATCGAAATTCTGGTTCCAAACTCCTTCGAAGAAGAGCGCATTAGCGTAAATTAGCGGTGTTAAGTTGTTAACTGCCCCTCGAGGAACAATTTCCTCTACGATTCCGTTCGTCCGTCT

>scaffold900_(220bp)-S900

GGTTGCTACAGGATTCAGCTACGTGGAGGACGAATCGTTGGTAGTTAGAACCGACTACGAGTCAGCAACTGATTTAACTACCTTGTTGAAGGCATTGTACAATGACAATGATGCCCTCCAGAAGAGCCCTCTCTATATCTTTGCCGAGTCTTATGGAGGAAAATTTGCTGTCACCCTTGGAGTTACCGCAGTTAAAGCCATCGAAGCAGGAGAGTTAAGG

**PMEs**

>Lus10031470_b3*b3_(196bp)-*LuPME79*

TCCCGATGGCCCACCAGTTCAACGCGATCACGGCTCAGAGCCGGACCGATCCGAATCAGAACACGGGGATATCGATCCAAAACTGTAGTATCAAGGCCGCGAAGGATCTTGCGGAGAGCAACGGAACGACTAGGTCTTACCTCGGCCGGCCGTGGAAGGCGTATTCGAGGACGGTGGTGATGAATTCGTACATCGC

>Lus10004720_b3*b2_(197bp)-*LuPME10*

GTTACATTCAATAGCGAAGAGATTTGGATCGATAGCGGCCCAAGACAGGAAATCGCCAGACGAGAAGACGGGCTTTGCATTCTTGAACTGTAAGGTGACTGGAACGGGCCAGCTCTACGTGGGCCGGGCCATGGGCCAGTACTCCAGGATTGTCTACTCACACACCTACTTTGATGACGTGGTTGCACACGGTGGAT

>G25305_a1*a1.2_(211bp)-*LuPME73*

GTTGACGTTTAGGAACACTGCCGGGCCGGCGAAGCACCAAGCAGTCGCCGTGAGAAACAGCGCCGACATGTCGGCGTTCTTCAACTGCAGCTTCGAAGGCTACCAGGATACACTATACGTACATTCCCTCCGCCAGTTCTACCGCGACTGTGACATCTACGGCACCATCGACTACATCTTCGGGAACGCGGCCGTCGTGTTCCAGAACTGC

>Lus10043035_a6*a6(220bp)-*LuPME105*

CGTTCGTGGGCTGCAGATTCCTGGGCGGACAGGACACTCTGTACGATCATTTCGGGAGGCATTATTACAAAGGTTGCTACATTGAAGGATCTGTGGATTTCATCTTCGGGAACGGCCTCTCCTACTTTGAGGTATGTATATTAATTTTTTGAATCAGAGAGAATATGTCGAATTGAAAGTGATGAATTTGGGGGTAAATCGTAAATGAAAGGGGTGTCAC

**Metabolism genes**

>Lus10016751_3.3F*3bR_(199bp)-acetolactate synthase-1

GTTCAAGAGCTGGCAACTATTCGGGTGGAGAATTTACCGGTGAAGATGATGCTGTTGAATAATCAGCACTTGGGTATGGTGGTACAGTGGGAAGATCGTTTCTACAAAGCGAATAGAGCTCACACATATCTAGGGGATCCAGCAAGGGAATCGGAGATATTCCCGAACATGCTGAAGTTTGCTGAAGGTTGTGGAATTC

>G24175_4F*4R_(206bp)-cyclic peptide

ACCTTGTCTCCTATTTCTGGAAAGGATGGCGGCCTCCGCAACCAGGAGGAGAGCGATGGTATGTTGGTCTTCCCCTTATTTATATTCGGCAAGGAAGGTAGTCAGGACAAGTATAATGGAGCAGCTGCCCTCCGCGACCAGGAGGAGAGCGATGGTATGTTGATCCCCCCCTTCTTTGTCATATTCGGCAAGGAAGGTTGTCAGGA

>Lus10029955_b.1F*bR_(208bp)-acetolactate synthase-2

ATGATACTGAACAACCAGCATTTGGGGATGGTGGTCCAGTGGGAGGACAGGTTTTACAAGGCGAACAGAGCGCATACGTTTCTGGGGAACCCGGCGGGGGAGGAAGGGGAGATTTTTCCGAACATGTTGAATTTCGCCGAGGCTTGTGGGATACCGGCGGCCAGGGTGAGTAAGATCGGCGAGGTTAGGGAGGGGATTCAGAGGATGT

>Lus10017825_aF*aR_(210bp)-UDP glucoronosyl/glucosyl transferase

ACCACAGCGGACTTATATTGCAAGACCCAGAACACTATTCCCAAACCTTTCGGAGAGTGGGTTCCGAAACCCTCACGGATCTCATCCGGAAACAGAGCGAATCNCGGCACCCTGTCCACTGTATAATTTACGATGCAAGTATGCCCTGGTTCCTGGACGTCGCCAAGCGGTTTGGGATTGTGGGAGCTGCATTTCTCACTCAGTCATGCG
