# Supplementary material for: Ion Torrent sequencing as a tool for mutation discovery in the flax (Linum usitatissimum L.) genome
Source: Plant Methods. 2015 Mar 14;11:19. doi: 10.1186/s13007-015-0062-x (PMC4363359; doi:10.1186/s13007-015-0062-x)
Supplement: Additional file 5: — Alignment of sequenced fragments of the pilot experiment. The number 1 over the alignment indicates position 1 for reference of mutations found. [file 13007_2015_62_MOESM5_ESM.docx]

**Additional file 5.** Alignment of sequenced fragments of the pilot experiment. The number 1 over the alignment indicates position 1 for reference of mutations found.

**Alignment F5*R5 – scaffold 20**

1

Bethune - F5*R5 scaffold 20 (1) CCGGTGTCTTCATTGTTGCGTCTTTCTCCGTCATGGTATTAGTCATGAAT

Macbeth - F5*R5 scaffold 20 (1) CCGGTGTCTTCATTGTTGCGTCTTTCTCCGTCATGGTATTAGTCATGAAT

30 65 Bethune - F5*R5 scaffold 20 (51) TTACTACTTTTTTCACTGCACATTCCATGACATAATCTGATTCTAACTGC

Macbeth - F5*R5 scaffold 20 (51) TTACCACTTTTTTCACTGCACATTCCATGACATAATCTGCTTCTAACTGC

Bethune - F5*R5 scaffold 20 (101) ATCGTTTTGTTAGAGTTTCTGGCTCGGATGGATTGTTAAATCATGGCCAC

Macbeth - F5*R5 scaffold 20 (101) ATCGTTTTGTTAGAGTTTCTGGCTCGGATGGATTGTTAAATCATGGCCAC

Bethune - F5*R5 scaffold 20 (151) TATTTTGGGCTCTCTTCATCAGTTTTGTCCTTGGCACTGCCTATTCAATC

Macbeth - F5*R5 scaffold 20 (151) TATTTTGGGCTCTCTTCATCAGTTTTGTCCTTGGCACTGCCTATTCAATC

Bethune - F5*R5 scaffold 20 (201) AATGTAAGTTCAGTTCGGCTCTATGTTCTTTCCGCAACCTGG---

Macbeth - F5*R5 scaffold 20 (201) AATGTAAGTTTTGTTCGGCTCTATGTTCTTTCCGCAACCTGGCTG

**Alignment F13*R13 – scaffold 411**

1

Bethune - F13*R13 scaffold 411 (1) ----GAGAAAAGCAAGACCAACCCCAATTCCTTCGTTAAGGAAACATACT

Macbeth - F13*R13 scaffold 411 (1) GTTAGAGAAAGGCAAGACCAACCCCAATTCCTTCGTTAAGGAAACATACT

Bethune - F13*R13 scaffold 411 (47) GGTCCTCTTCGCTGGTCATAAACGGTACACGGACCGATCCATCATTCAGT

Macbeth - F13*R13 scaffold 411 (51) GGTCCTCTTCGCTGGTCATAAACGGTACACGGACCGATCCATCATTCAGT

Bethune - F13*R13 scaffold 411 (97) AGGTGAAAATCATAGCGTTTAGTTGCCGATGCATCGAAATTCTGGTTCCA

Macbeth - F13*R13 scaffold 411 (101) AGGTGAAAATCATAGCGTTTAGTTGCCGATGCATCGAAATTCTGGTTCCA

120

Bethune - F13*R13 scaffold 411 (147) AACTCCTTCGAAGAAGAGCGCATTAGCGTAAATTAGCGGTGTTAAGTTGT

Macbeth - F13*R13 scaffold 411 (151) AACTCCTTCGAAGAAGAGCGCATTAGCGTAAATTAGCGGTGTTAAGTTGT

Bethune - F13*R13 scaffold 411 (197) TAACTGCCCCTCGAGGAACAATTTCCTCTACGATTCCGTTCGTCCGTCTA

Macbeth - F13*R13 scaffold 411 (201) TAACTGCCCCTCGAGGAACAATTTCCTCTACGATTCCGTTCGTCCGTCTA

**Alignment F23*R25 – scaffold 900**

Bethune - F23*R25 scaffold 900 (1) GCCGACCTACTGTTCGTGGTATAATTTTCTAACTAGAAAACATTTTCATC

Macbeth - F23*R25 scaffold 900 (1) GCCGACCTACTGTTCGTGGTATAATTTTCTAACTAGAAAACATTTTCATC

Bethune - F23*R25 scaffold 900 (51) CAAAAAAATTCATAATCTTTCTGTTAATCGATGTTGATGGAAATATCAAT

Macbeth - F23*R25 scaffold 900 (51) CAAAAAAATTCATAATCTTCCTGTTAATCGATGTTGATGGAAATATCAAT

1 28

Bethune - F23*R25 scaffold 900 (101) TATTCTCAGGATAGTCCGGTTGCTACAGGATTCAGCTACGTGGAGGACGA

Macbeth - F23*R25 scaffold 900 (101) TATTCTCAGGATAGTCCGGTTGCTACAGGATTCAGCTACGTGGAAGACGA

Bethune - F23*R25 scaffold 900 (151) ATCGTTGGTAGTTAGAACCGACTACGAGTCAGCAACTGATTTAACTACCT

Macbeth - F23*R25 scaffold 900 (151) ATCGTTGGTAGTTAGAACCGACTACGAGTCAGCAACTGATTTAACTACCT

121

Bethune - F23*R25 scaffold 900 (201) TGTTGAAGGCATTGTACAATGACAATGATGCCCTCCAGAAGAGCCCTCTC

Macbeth - F23*R25 scaffold 900 (201) TGTTGAAGGCATTGTACAATGACAATGATGCCCTCCAAAAGAGCCCTCTC

Bethune - F23*R25 scaffold 900 (251) TATATCTTTGCCGAGTCTTATGGAGGAAAATTTGCTGTCACCCTTGGAGT

Macbeth - F23*R25 scaffold 900 (251) TATATCTTTGCCGAGTCTTATGGAGGAAAATTTGCTGTCACCCTTGGAGT

Bethune - F23*R25 scaffold 900 (301) TACCGCAGTTAAAGCCATCGAAGCAGGAGAGTTAAGGCTCCAACTCGGAG

Macbeth - F23*R25 scaffold 900 (301) TACCGCAGTTAAAGCCATCGAAGCAGGAGAGTTAAGGCTCCAACTCGGAG

Bethune - F23*R25 scaffold 900 (351) GTTAAGAAA

Macbeth - F23*R25 scaffold 900 (351) GTTAAGAAA

**Primers of the amplicons used for Ion torrent: black bar**

**Primers used for sequencing: red bar**

**Expected mutations: black outline**

**Unreferenced mutations: red outline**
