# Supplementary material for: Ion Torrent sequencing as a tool for mutation discovery in the flax (Linum usitatissimum L.) genome
Source: Plant Methods. 2015 Mar 14;11:19. doi: 10.1186/s13007-015-0062-x (PMC4363359; doi:10.1186/s13007-015-0062-x)
Supplement: Additional file 6: — Ion Sphere Particles (ISPs) and read identification summary. The data is given for two technical replicates runs (A and B) of PMEs using the Ion Torrent PGM. [file 13007_2015_62_MOESM6_ESM.docx]

**Additional file 6.** Ion Sphere Particles (ISPs) and read identification summary. The data is given for two technical replicates runs (A and B) of PMEs using the Ion Torrent-PGM.


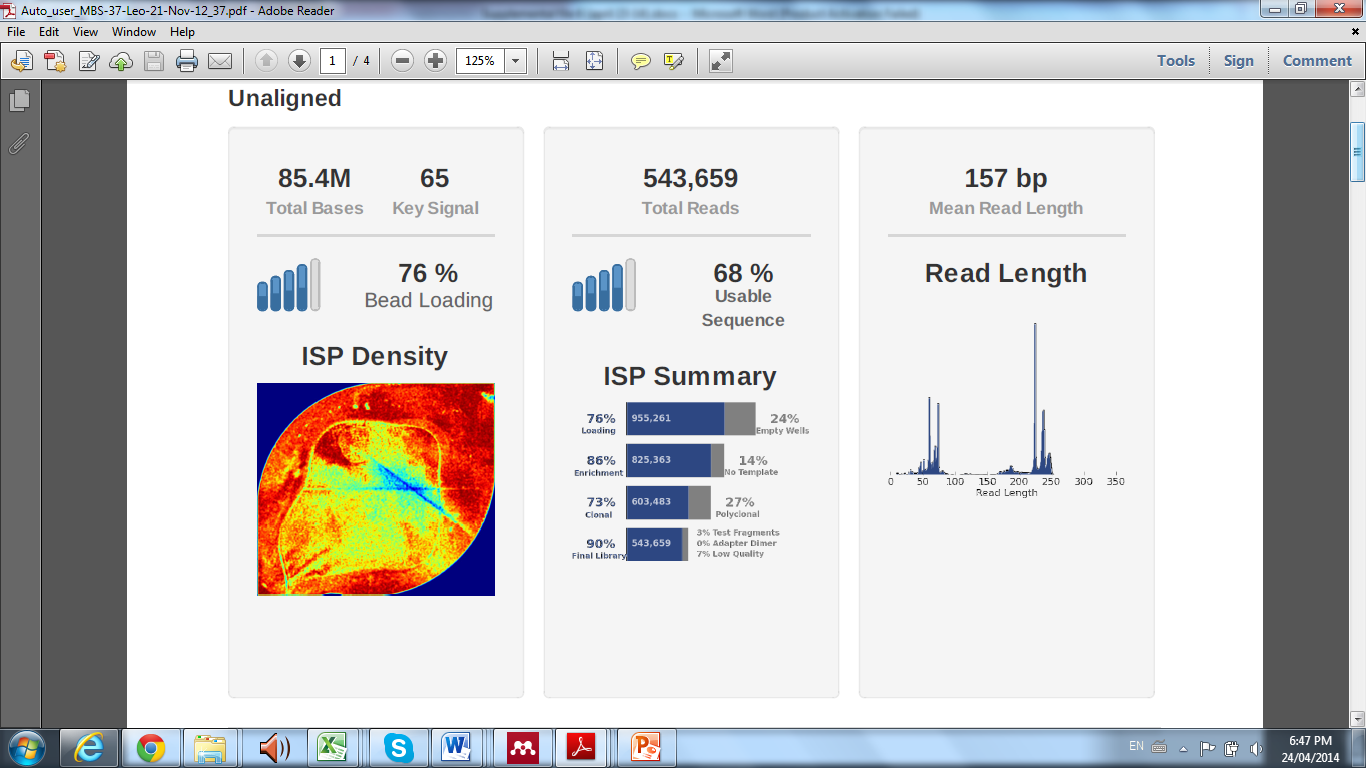

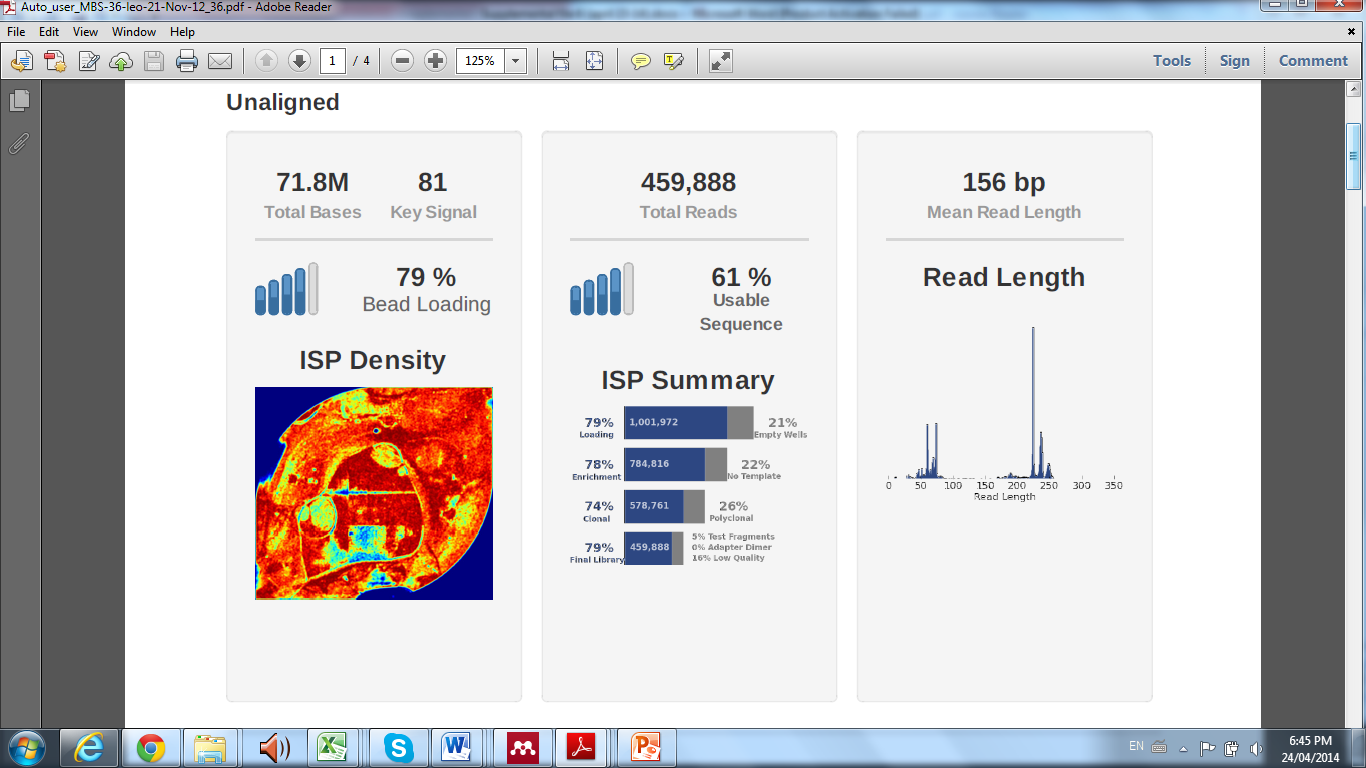


A

B
