# Supplementary material for: Ion Torrent sequencing as a tool for mutation discovery in the flax (Linum usitatissimum L.) genome
Source: Plant Methods. 2015 Mar 14;11:19. doi: 10.1186/s13007-015-0062-x (PMC4363359; doi:10.1186/s13007-015-0062-x)
Supplement: Additional file 7: — Average read count and dispersion among the mapped reads for two replicate sequencing runs of PME genes. Statistics are given for the 28 pools for all mapped reads or for each individual gene. [file 13007_2015_62_MOESM7_ESM.docx]

**Additional file 7**. Average read count and dispersion among the mapped reads for two replicate sequencing runs of PME genes. Statistics are given for the 28 pools for all mapped reads or for each individual gene.

| **Reads** | **Replicate** | **Average** | **Mean deviation** | **Standard deviation** | **Coefficient of variation** |
| --- | --- | --- | --- | --- | --- |
| **Mapped reads** | 1 | 9906.0 | 1939.3 | 2461.8 | 24.9 |
|  | 2 | 12236.5 | 2499.4 | 3175.1 | 25.9 |
| **Lus10031470 (*LuPME79*)** | 1 | 1362.6 | 464.2 | 693.8 | 50.9 |
|  | 2 | 1700.7 | 485.2 | 715.0 | 42.0 |
| **Lus10004720 (*LuPME10*)** | 1 | 3277.0 | 1199.8 | 1534.1 | 46.8 |
|  | 2 | 3138.9 | 1147.7 | 1478.6 | 47.1 |
| **G25305 (*LuPME73*)** | 1 | 3365.8 | 887.6 | 1279.2 | 38.0 |
|  | 2 | 5136.9 | 1387.4 | 1977.0 | 38.5 |
| **Lus10043035**  **(*Lu PME105*)** | 1 | 1900.6 | 820.7 | 1092.9 | 57.5 |
|  | 2 | 2260.1 | 907.0 | 1227.5 | 54.3 |
