# Supplementary material for: Ion Torrent sequencing as a tool for mutation discovery in the flax (Linum usitatissimum L.) genome
Source: Plant Methods. 2015 Mar 14;11:19. doi: 10.1186/s13007-015-0062-x (PMC4363359; doi:10.1186/s13007-015-0062-x)
Supplement: Additional file 8: — Python script algorithm description. The methodology, results and discussion using this script are presented. [file 13007_2015_62_MOESM8_ESM.docx]

**Additional file 8.** Python script algorithm description. The methodology, results and discussion using this script are presented.

**Methodology**

Although CLC allows creating a workflow to map and create frequency tables for each position and gene examined, the large amount of data created had to be analyzed by hand to create the graphs and find the intersecting pools indicative of an SNV. We decided to create a custom algorithm that simplified this downstream process where raw .txt files are uploaded from CLC to the custom graphical user interface (GUI); files are then automatically parsed and ready for statistical analysis. Statistical parameters can be adjusted to find the variants under varying stringencies.

First the script takes all of the parsed data and looks at the frequencies at every position and pool intersection (rows missing data are deleted). The remaining frequencies are assumed to be both a signal and noise simultaneously, and a signal to noise ratio is calculated to pick out the outstanding points in each group using the following formula:

1. $\frac{S}{N}$=$\frac{X}{\sqrt{\frac{\sum_{i=0}^{n} {(X-\bar{X})}^{2}}{n}}}$;

where S is the signal representing a given frequency, N is the noise of that frequency compared to other surrounding frequencies; X is the frequency that is given by the intersection of a pool and position in a given gene, $\bar{X}$ is the average frequencies of all pools in a given position, and n is the total population of points in a given position (maximum of 28 since there are only 28 pools). Converting each frequency into a signal to noise ratio better separated the mutations when the process was compared to looking at just the raw frequencies.

Next the script uses four statistical parameters to decide whether a point should be kept. First, confidence intervals are used within each group (group A is pools 1 to 8, group B is pools 9 to 20, and group C is pools 21 to 28 – see Figure 2). These are constructed by using the following formula(s):

1. C.I=$\bar{X}\pm$ $\frac{(\frac{Var 1}{200})\bigcap(n-1)\times\sigma}{\sqrt{n}}$;

where C.I is the confidence interval, $\bar{X}$ is the mean signal to noise ratio with a group, var1 is the confidence interval chosen within the GUI (ie. 99%),$\sigma$ is the standard deviation of the signal to noise ratios within a group, and n is the population within a group (a maximum of 8 pools in group A and C, and a maximum of 12 pools in group B). The expression $(\frac{Var 1}{200})$ takes the chosen confidence interval in the GUI and converts it on an alpha value; the n-1 expression is the degrees of freedom therefore the expression $(\frac{Var 1}{200})\bigcap(n-1)$ is used to find a critical value located in a referenced t-table by finding the intersection between the degrees of freedom within a group and the alpha value. A case where there is only 1 pool in a group (degrees of freedom is zero) is solved by simply reporting the only pool in the group and bypassing the statistical algorithm completely. At this point the script looks through all pools in a group and discards the ones that fall below the upper confidence interval; lower confidence intervals are not used because we are only interested in outliers above the baseline noise.

Next, the script looks at the mean signal to noise ratios of the entire population at a given position and discards points that are below a chosen standard deviation (Var 2 in the GUI). For example, if 3 standard deviations are chosen then only points that are past 3 standard deviations are kept.

Then, a cutoff point is chosen by the user (Var 3 in the GUI) in order to discard extreme outliers. For example, if 6 standard deviations are chosen, all points greater than or equal to 6 standard deviations will be discarded. This is done because extreme outliers have been found to be related to a sequencing error.

The overall conditions that must be met in order for coordinates to be reported at the end of the script are:

1. Var 2 and Var 1. $\leq$ $\frac{S}{N}$ $\leq$ Var 3.
2. n $\geq$0; there must be at least 1 or 2 pools from each group depending on what condition is chosen in the “minimum pools” box in the statistics tab found in the GUI (Variable 4, separate from the previous mathematical formulas).

There are some cases where more than one pool meets all of the conditions and thus more than one pool is reported for a group. For example, pools 1 and 3 (A1 and A3) may have very close frequencies that meet all conditions so both are reported. In such cases the script reports all possible combinations of selected points which point to one or more individuals with potential mutations. The number of potential individuals will vary according to the stringency of the statistics used.

To start off, a stringency level of 95% C.I., 3 S.D., and a cutoff of 6 S.D. should be used to avoid overwhelming numbers of suggested individuals and false positives. This automated procedure was applied to the metabolism related genes as a way to compare the results with the visual analyses previously performed.

**Results and discussion.**

For our analysis we used four metabolism-related genes looking at two mutation types (C to T or G to A) and 28 pools. These analyses therefore yielded a total of 4*2*28 = 224 files. If each file has an average of 200 positions, that’s 44800 positions for analysis. Working through several genes and creating graphs for each mutation types can take months of full time work whereas using the script reduced the work down to a few hours. The graphical analysis without using any statistical analysis has merit. You can visually see intersecting pools that appear above the noise of all the other pools; a mutated individual is thus easier to visually and the analysis can be made at a per-position basis. You can also expand the Y-axis and find intersecting pools that are closely grouped within the noise (this is how we found the stop codon). The downside is mainly the amount of time it takes to perform the analysis and that choosing the intersecting pools is more subjective and not quantifiable. Using the statistics embedded in the GUI is much faster but it has the potential exclude points that may be important depending on the stringency parameters. Overall the parsing and statistical analysis behind the script methodology allow for a high-thoughput way of analyzing large sets of data. However, we saw that many points that we found visually could not be detected using the program, which is due in part to the variability of data. The recommended technical and methodological aforementioned adjustments should improve the detection quality and allow for a future detection relaying exclusively on the script.
